# Probiotics in IBD: Evidence and Perspectives on Patient Health and Disease Management

**DOI:** 10.3390/ijms26189065

**Published:** 2025-09-17

**Authors:** Emília Hijová

**Affiliations:** Center of Clinical and Preclinical Research-MediPark, Pavol Jozef Šafárik University in Košice, 04011 Košice, Slovakia; emilia.hijova@upjs.sk

**Keywords:** probiotics, ulcerative colitis, Crohn’s disease

## Abstract

Inflammatory bowel disease (IBD) includes two distinct diseases: ulcerative colitis (UC) and Crohn’s disease, affecting people worldwide regardless of age and gender. It appears that a combination of many factors, primarily genetic background, environmental, host immune response, and a state of reduced microbial diversity are associated with IBD. Gut modulation by probiotics application represents one of the potential strategies for the prevention or treatment of IBD. The gut microbiota has the ability to influence host physiology either directly or through microbial metabolites. This review summarizes human randomized clinical trials that evaluate the usefulness of various probiotics in relation to the treatment, prevention, and maintenance of disease remission.

## 1. Introduction

Inflammatory bowel disease (IBD) represents a significant public health challenge worldwide, and is caused by a combination of host genetics, immunological imbalance, microbial and environmental factors [1]. IBD affects various parts of the gastrointestinal tract, with persistent inflammation of the gastrointestinal tract leading to a shortened lifespan [2,3]. IBD affects nearly 2 million people in Europe and 1.6 million people in the United States. Epidemiological studies have shown an increasing incidence of IBD in developing regions such as South America, Asia, Africa, and Eastern Europe [4,5]. Ulcerative colitis (UC) and Crohn’s disease (CD) are the two main subtypes of IBDs. This multifactorial condition results from interactions between genetic, environmental, immune, psychological, and microbial factors. These various factors lead to the disruption of mucosal permeability, also known as “leaky gut”, and increased immune activity in the intestinal mucosa. Severe complications of inflammatory conditions include anemia, malnutrition, infections, and an increased risk of colon cancer. Certain beneficial microbes, known as probiotics, have been identified as promising therapeutic agents for inflammatory conditions such as IBD [6]. Probiotics are viable microorganisms that confer advantageous health effects on the host. The promising role of probiotics for IBD is demonstrated by several pre-clinical and clinical studies [7,8]. These outcomes are the results of variable mechanisms, and the production of microbial metabolites are known to be trophic factors—short-chain fatty acids (SCFAs), most notably butyrate, decrease the level of immunoglobulin A (IgA) and cause an upregulation of defensins and mucin-2 expression, and reduce the production of pro-inflammatory cytokines [8,9].

This review explores mechanisms by which probiotics can restore the balance of gut microbiota, increase short chain fatty acids (SCFAs) production, increase the integrity of the gut mucosal layer, and reduce inflammation by degrading histamine, which are all essential in the treatment of IBD. Given the growing prevalence of IBD and the limitations of current treatments, this topic is timely and of great clinical relevance.

## 2. Intestinal Microbiota in Management of IBD

Various diseases are associated with inflammatory processes asa natural defense responses mediated by active immune cells. Dysregulated, innate, and adaptive immune responses may be the result of an interaction between genetic abnormalities, environmental factors (diet, lifestyle, andstress), permeability of gut barrier, and altered intestinal microbiota in IBD [10,11]. IBD includes conditions, such as Crohn’s disease and ulcerative colitis, which are chronic inflammatory diseases of the digestive tract. UC mostly affects the colon and rectum, while CD can manifest in the gastrointestinal tract [12].

The increasing incidence of IBD in newly industrialized countries has been attributed to significant dietary changes, such as increased intake of refined sugars and processed foods and decreased consumption of plant-based foods [13].

Microbiota play a significant role in the development, progression, and treatment of IBD. Gut dysbiosis is characterized by a reduction in beneficial microbiota and overall microbial diversity, as well as an excessive amounts of potentially harmful organisms. The dysbiosis in the gut is accompanied by many adverse impacts including dysfunctional mucosal epithelial cells, impaired of gut immune homeostasis, and uncontrolled gut inflammation. Studies have shown that gut dysbiosis is an important pathogenic factor in the development of IBD; the number of bacteria associated with inflammation in IBD patients is increased in comparison to healthy individuals [14,15].

The numbers of beneficial bacteria such as *Bifidobacterium longum*, *Faecalibacterium prausnitzii*, *Akkermansia muciniphila, Roseburia intestinalis*, and *Eubacterium rectale* are significantly lower in patients with CD and UC compared to harmful bacteria, which are significantly increased [16,17]. The main pathogenic microbes that cause IBD are *Escherichia coli (E. coli)*, *Bacteroides fragilis*, *Fusobacterium varium*, *Fusobacterium nucleatum*, *Helicobacter species*, and *Campylobacter concisus. Clostridioides difficile*, a Gram-positive anaerobic bacteria causing diarrhea and colitis, and *B. fragilis* stimulate goblet cells and dendritic cells (DCs) to release interleukin-10 (IL-10) and transforming growth factor-beta (TGF-β), which can increase the regulatory T-cells (T--regss) population [18,19]. Increased amounts of *Proteobacteria* are generally considered characteristic of gut dysbiosis [20].

The role of the microbiome in IBD is supported by the identification of gene mutations associated with microbiome-immune interactions in patients with IBD. Genome-wide studies have identified over 200 genetic loci that are associated with IBD.

Genes associated with an increased risk of IBD include nucleotide-binding oligomerization domain containing 2 (NOD2), immunity-related quanosine triphosphatase family M (IRGM), autophagy-related 16-like 1 (ATG16L1), protein tyrosine phosphatase non-receptor type 2 (PTPN2), interleukin-10 receptor subunit alpha (IL-10R1), leucine-rich repeat kinase 2 (LRRK2), interleukin-23 receptor (IL-23R), interleukin-10 (IL-10), interleukin-10 receptor subunit beta (IL-10R2), hepatocyte nuclear factor 4-alpha (HNF4-α), and cadherin 1 (CDH1). Incorporating a genetic panel that covers key genes could significantly improve the diagnosis and assessment of IBD [21,22].

The first line of defense of the internal environment against the external environment is the gut barrier, an important aspect of IBD, which contains intact and tightly attached epithelial cells.

The gut is exposed to many factors (food antigens, resident microbiota, pathogenic organisms, etc.). The role of the barrier is, firstly, to prevent the penetration of useless antigens, and secondly, the passage of nutrients through the intestinal wall. In IBD, this barrier is disrupted, causing its increased permeability. The direct causes of increased permeability are unknown. The regulation of gut epithelial permeability is associated with alterations in CARD15/NOD2, OCTN1, CD1H, and C1ORF106 genes [23].

Homeostasis of the mechanisms involved in maintaining a functional intestinal barrier is a key factor in the initiation and progression of IBD. The disruption of the functionality and integrity of the intestinal barrier occurs due to excessive activation of immunocompetent cells in the mucosa (macrophages, neutrophils, and dendritic cells), which subsequently produce pro-inflammatory cytokines such as tumor necrosis factor-alpha (TNF-α), interferon-gamma (IFN-γ), interleukin-1 beta (IL-1β), and interleukin-6 (IL-6), disrupting tight junction proteins (TJPs) between epithelial cells, thereby contributing to the formation of ulcerations and the degradation of the GIT mucosa [24]. Disruption of the gut barrier in patients with IBD alters the expression of tight junction proteins and may exacerbate IBD. The expression of the TJP can be modulated by pro-inflammatory cytokines such as interleukin-13 (IL-13), TNF family members, andIFN-γ, and increase epithelial permeability through disruption of epithelial junctions [25,26]. The products containing *Faecalibacterium prausnitzii* improved the tightness of the gut barrier in mice with DSS-induced colitis [27]. The gut barrier interacts with innate immune cells, including innate lymphoid cells (ILCs), macrophages, natural killer (NK) cells, neutrophils, and dendritic cells.

Intestinal macrophages are key immune cells in the maintaining intestinal immune homeostasis and have a role in the pathogenesis of inflammatory bowel diseases. Dharmasiri et al. [2] demonstrated for the first time (and confirmed by quantitative polymerase chain reaction and immunohistochemistry) that intestinal macrophages from IBD patients display both M1 and M2 features, as recently described for tumor-associated macrophages that influence key pathways for IBD pathology, represented by markers such as matrix metalloproteinase-12 (MMP12), CXCL9, and CD40. The design of future immunotherapies in IBD is to reprogram intestinal macrophages toward M2 which may not only fail to reduce M1 activation but may also potentially exacerbate fibrotic and granuloma formation in CD. The new drugs should be able to target and deactivate both M1/M2 pathways, thereby restoring the ability of macrophages to balance the immune homeostasis in the intestinal lining and combat xenobiotics.

The immunological state of the organism is an important factor in the development of IBD. The most important cells appear to be helper lymphocytes Th (Th1, Th2,and Th17) and T-regulatory (T-reg) cells. The polarization between Th1 or Th2 depends on the cytokine profile produced as an effect of the microorganism which is recognized by MAMPs (microbe-associated molecular patterns). An imbalance between Th17 cells (promote tissue inflammation) and T-reg cells (suppress autoimmunity in IBD), which are differentfrom CD4+ T-cells, contribute to IBD [28]. It has been described that CD is connected with Th1 cells skewing, while UC with Th2 cells. In UC, Th2 cells activity increases due to the release of interleukin-4 (IL-4), IL-5, IL-6, and IL-13, which are associatedwith allergic reactions- and parasitic worms. In CD, Th1 cells produce tumor necrosis factor beta (TNF-β), interferon gamma (IFN-γ), and IL-2, which are associated with intracellular bacteria and virus killing. For both diseases, activity of the Th17 cells seems to be important and situated on the gut’s mucosa. Th17 cells produce several pro-inflammatory cytokines such as IL-17, IL-17A, IL-17F, IL-22, tumor necrosis alpha (TNF-α), and the chemokine CCL20. IL-17 is involved in defense against extracellular pathogens. Th1 cells and Th17 cells activity increases due to the production of TNF-α, IFN-γ, IL-2, IL-12, IL-18, and IL-23, which are produced by antigen-presenting cells (APCs) and macrophages [24].

The pro-inflammatory cytokine TNF-α, produced by macrophages, monocytes, and T cells, has a key role in the pathogenesis of IBD. The transfer of immune cells, particularly T cells, to the intestine is essential for initiating and maintaining immune responses, making it a critical factor in the development of IBD [18]. TNF-α and IL-6 trigger inflammation in IBD and act together with interleukin 1-beta (IL-1β).

Mucin production in the colonic mucosa in response to inflammation is regulated by many factors, such as growth factors, microbes, pro-inflammatory cytokines, toxins, and neuropeptides. Interleukin-10 and its receptors are expressed on various immune cells, allowing IL-10 to efficiently regulate innate and adaptive immune responses. Interleukin-23 plays a pivotal role in disease development, and the development of selective IL-23-targeted therapies represents key components of personalized medicine [29]. Probiotic product supplementation (Jamieson Laboratories, Canada) significantly reduces IgA levels, while IgM and IgG levels are not significantly reduced [30]. Similar results were also reported in the study using the probiotic mixture VSL#3 on chronic colitis in C57BL/6 mice induced by dextran sulfate sodium. They observed that VSL#3 treatment significantly reduced levels of IgM, IgG, and IgA in colon mucus and thedown-regulated the number of T follicular helper (Tfh) cells in mesenteric lymph nodes, thereby possibly alleviating colitis [31].

## 3. Probiotics and Their Beneficial Potential in IBD

Control of inflammatory pathways can be achieved through various strategies such as pharmaceutical treatments, lifestyle modifications, and dietary adjustments. The long-term use of pharmacological interventions in current IBD treatments, such as drugs, corticosteroids, amino-salicylates, anti-TNF agents, anti-integrins, calcineurin inhibitors, immunosuppressants, biologics, and antibiotics, is accompanied by side effects, including digestive problems, immune suppression, diabetes, kidney damage, hypertension, weight gain, and an increased risk of infections. Surgical intervention treatment of IBD can lead to complications such as intestinal perforation, pelvic infection, and massive bleeding [32]. The relationship between gut microbial dysbiosis and the development of IBD provides a new available treatment option through various microbial therapies, such as probiotics [33] prebiotics [34], fecal microbiota transplantation [35], and nutritional supplements [36].

Probiotics are defined as live microorganisms that benefit the health of the host by favorably altering the gut microflora after oral intake in adequate amounts [37]. Complementary therapy is often the most widely used adjunctive therapy for gastrointestinal diseases and are often recommended by physicians [38,39].

Probiotics, or beneficial live microorganisms, have shown promise in prevention and treatment of inflammatory bowel diseases by promoting balance in the intestinal microbiota and increasing its diversity and composition, thereby contributing to the prevention or reduction of dysbiosis [40]. Restoring the balance of microbiota can modulate the local immune response, reduce inflammation, and strengthen the integrity of the intestinal barrier. Modulated immune responses promote the production of anti-inflammatory cytokines as well as short chain fatty acids.

Probiotics have the potential to maintain a healthy intestinal environment through interaction with the immune system and microbiota, which could be essential in preventing the development of IBD [41].

Patients with IBD experience significant disruptions in the composition of the intestinal microbiota, characterized by a significant loss of microbial diversity and the development of dysbiosis [42]. Depletion of beneficial commensal bacteria that are integral to maintaining intestinal health, such as *F. prausnitzii*, *A. muciniphila, Bifidobacterium*, and *Lactobacillus* spp., and the over-representation of pathobionts, such as *E. coli* (especially adherent-invasive strains) and *Clostridioides difficile*, is a consistent feature of IBD [43].

These changes affect key functions of the gut microbiota, including the synthesis of beneficial metabolites such as SCFAs, disruption of the intestinal barrier, increased permeability, and promotion of the translocation of microbial products such as lipopolysaccharides (LPSs), which exacerbate mucosal inflammation and immune activation [44]. Acetate, propionate, and butyrate are the main SCFAs, of which butyrate is essential for maintaining energy metabolism, epithelial repair, and anti-inflammatory signaling [45].

Probiotic interventions targeting the gut microbiota may help restore microbial homeostasis and reduce systemic effects in patients with IBD. Histamine-degrading probiotic bacteria such as *Lactiplantibacillus plantarum* and *Limosilactobacillus reuteri* have shown the potential to reduce the amount of histamine in the intestine, reduce excessive inflammation, and prevent intestinal permeability [46,47]. Regulation of histamine receptors by some antihistamine drugs can block histamine receptors, modulating their activity, which reduces their pro-inflammatory effects [48].

Animal and human studies show that probiotics can have a key and preventive effect on the modulating immune and inflammatory mechanisms; however, the exact mechanisms are not yet well defined [49,50,51].

## 4. Probiotic Molecular Mechanism of Action in IBD

Taking probiotics provides an alternative that restores the balance of intestinal microbiota and shifts the balance from a pro-inflammatory state to an anti-inflammatory one.

These are the most commonly available strains used as probiotics all of which have beneficial health effects applied alone or in combination [52,53]: (1) the *Bifidobacterium* species, (2) *Enterococcus faecium*, (3) the *Lactobacillus strains*, (4) *Saccharomyces boulardii*, (5) the *Bacillus* species, and (6) *Pediococcus*. 

The general mechanism the action of probiotics focuses on two main aspects:

1. Altering the composition and function of the gut microbiome;
2. Supporting the physiology and immunobiology the intestinal mucosa to promote an anti-inflammatory response and facilitate wound healing.

The beneficial effect of probiotics includes the following molecular mechanisms:

(a) Production of SCFAs, especially butyrate and stimulatory signaling proteins;
(b) Formation of immunoglobulin A;
(c) Reduced production of pro-inflammatory cytokines;
(d) Induction production of anti-inflammatory cytokines;
(e) Increased expression of mucin-2;
(f) Increased autophagy and upregulation of defensing [8,54].

The mechanisms of action of probiotics include increased adhesion of healthy probiotic bacteria to intestinal mucosa, inhibition of pathogens adhesion to the mucosal surface, competitive exclusion of pathogenic microorganisms, enhancement of the epithelial barrier function, and modulation of the immune function. The beneficial mechanisms of probiotics like *L. reuteri*, *B. longum* and *E. coli* Nissle 1917 are highly dependent on the specific strain. These mechanisms include direct interactions with pathogens, modulation of the host immune system, maintenance of the intestinal barrier, and production of beneficial metabolites, all of which vary significantly by strain. Mechanistic studies show that *L. reuteri*, *B. longum*, and *E. coli* Nissle 1917 exhibit strain-specific effects against pathogens and on the gut barrier through different mechanisms. For instance, *L. reuteri* can produce metabolites and modulate host immunity to support the gut barrier, while *B. longum* and *E. coli* Nissle lysates can inhibit pathogen growth and alter host cell interactions, with *E. coli* Nissle 1917 also potentially enhancing some pathogens’ growth or protecting against others’ toxic effects [55]. The probiotics promote beneficial effects through activity of microbial enzymes. Of interest is the inhibition of β-glucuronidase activities originating from harmful bacteria. These bacteria hydrolyze glucuronidated metabolites via β-glucuronidase activity in the intestinal lumen, leading to formation of toxic metabolites and intestinal damage. The study suggests probiotic supplements containing *L. plantarum* LS/07 for the decrease in β-glucuronidase activity associated with colon carcinogenesis [56].

Probiotics facilitate microbial diversity through competitive exclusion; probiotics compete with dysbiotic and pathogenic species for the receptor present in the gastrointestinal tract. Although regulatory elements and their associated specific pathways are largely unknown, some of the proposed mechanisms include the creation of an acidic environment to reduce competition, competing for nutritional sources, and producing of bacteriocins or bacteriocin-like substances for elimination of pathogens.

Probiotics and SCFAs maintain gut health. The gut has a delicate balance between the beneficial bacteria that release vitamins and harmful microbiota that secrete toxic substances. Probiotics and fibers contribute to the stabilization of beneficial microbiota which, in the colon, ferments proteins and fibers to produce SCFAs. Short chain fatty acids are the key signaling molecules in enterocytes and are an important source of their energy; absorbed SCFAs also stimulate cell surface receptors in several other tissues via entry into the systemic circulation. By increasing the production of SCFA, they can lower the pH of the intestinal environment, thereby inhibiting the growth of potentially pathogenic microorganisms. SCFAs promote intestinal secretion of the hormones such as polypeptide YY (PYY) and glucagon-like peptide 1 (GLP-1) and increase satiety by interacting with G protein-coupled receptor (GPR) 41 and GPR43 [57]. SCFAs interact with GPR43 on adipose tissues and contribute to the reduction in the fat deposits, leading to reduced lipolysis and inflammation and increased adipogenesis and leptin release. On the other hand, SCFAs could also enhance the activation of peroxisome proliferator–activated receptor (PPAR)-γ-mediated adipogenesis possibly via GPR43. In terms of immunomodulation, it is believed that propionate and butyrate reduce the secretion of pro-inflammatory cytokines and chemokines and reduce inflammation. Another mechanism involves promoting insulin sensitivity and reducing lipid accumulation through beta-oxidation by SCFAs via activation of AMP-activated protein kinase by SCFAs in muscles [57].

Some probiotics increase the integrity of the mucosal barrier, thereby normalizing intestinal permeability [58]. The effects of probiotics are varied and depend on the species and dose, as well as on their interaction with the host. Some exhibit direct antibacterial effects through the production of substances such as bacteriocins, hydroperoxides, lactic acid, and defensins. Probiotics can modulate production of immunoglobulins and pro-inflammatory cytokine by releasing cell wall fragments or DNA in the intestinal lumen. They also regulate the excessive activation of the nuclear factor kappa beta (NFκB) enhancer of the activated B cells pathway, reduce the production and secretion of pro-inflammatory cytokines such as IL-8, TNF-α, IFN-γ, and induce the production of anti-inflammatory cytokines such as IL-10 and TGF-β [59].

Probiotics suppress intestinal inflammation through several mechanisms targeting toll-like receptors (TLRs) signaling, including downregulation of TLR expression and secretion of metabolites that inhibit extracellular pro-inflammatory cytokines (TNF-α) or through the inhibition of NF-κB responsible for inflammatory cytokine expression in enterocytes. Other mechanisms by which probiotics suppress inflammation include the suppression of IL-12 production by the host cells [60] and enhancement of epithelial barrier functions supporting TJP formation [61].

## 5. Randomized Clinical Trials on the Use of Probiotics in Ulcerative Colitis

The Pubmed and Scopus databases were used to summarize randomized clinical trials in humans (children and adults) on the use of probiotics in ulcerative colitis [Table 1] and Crohn’s disease [Table 2] from 1997 to 2023.

More differentiated results are noticeable within patients-based studies. The most intensively studied bacterial strain among the Gram-negative microorganisms is a non-pathogenic strain of *E. coli*, *E.coli* Nissle 1917, which has been applied in six randomized clinical trials in patients with UC with different results. A study in patients with mild to moderate active UC reports the safety and efficacy of *E. coli* Nissle 1917 in achieving clinical responses and endoscopic remission [62]. Peterson et al. [63] found no benefit in the *E.coli* Nissle 1917 as an add-on treatment to conventional therapies for active UC. An enema with 40 mL of *E. coli* Nissle 1917 appears to be the most promising in the study by Matthes et al. [64]. In a study of 116 patients with active UC, the effect of 1 week of gentaminine treatment followed by *E. coli* Nissle 1917 treatment was equivalent to mesalazine treatment in inducing remission of UC [65]. Three large RCTs compared *E. coli* Nissle 1917 with mesalazine but without significant differences in relapse rates or side effects [65,66,67]. One pediatric trial confirmed that maintenance treatment of UC with the probiotic *E. coli* Nissle 1917 is effective, even in young patients [68].

The combination of nine *Lactobacillus* and five *Bifidobacterium* species in a probiotic product (Jamieson Lab., Canada, N8W5B5) has also been shown to induce remission in patients with ulcerative colitis, reduce levels of biochemical parameters and increase levels of hematological parameters [30], and improve quality of life (social, bowel, emotional) in patients with mild to moderate ulcerative colitis [69]. Lactocare^®^ (Zist Takhmar, Iran), a synbiotic product with two strains of *Bifidobacterium* and four strains of *Lactobacillus* and *Streptococcus thermophilus*, demonstrated a significant decreasein the simple clinical colitis activity index in the treatment group from 6.54 to 4.65 (*p* < 0.017) and in the placebo group from 5.7 to 5.21 (*p* < 0.17) [70]. A probiotic cocktail containing *Enterococcus faecalis*, *Bifidobacterium longum*, and *Lactobacillus acidophilus* improved the composition of the microbiota and reduced the level of inflammatory markers in patients with IBD (31 patients with ulcerative colitis and 9 patients with Crohn’s disease), as demonstrated by Fan et al. [71]. In patients with mild to moderate active UC treated with *Lactobacillus acidophilus*, *Lactobacillus plantarum*, *Lactobacillus rhamnosus*, *Lactobacillus bifidus*, *Lactobacillus casei*, and *Bifidobacterium infantis*, disease activity as assessed by the Truelove and Witts scale and the histological index as assessed by the Gupta index improved [72]. The multi-strain probiotic was superior to the placebo in reducing fecal calprotectin levels, but with no significant differences in IBD quality of life questionnaire scores between the two groups, as demonstrated by Bjarnason et al. [73].

Bifid Triple Viable (BTV) administered with mesalazine was more effective than mesalazine alone in reducing clinical symptom scores, the colon mucosa inflammation score, IL-1β expression, and in increasing expression of IL-10 and IgA in colon mucosa, as showed Li et al. [74]. Similar results have been shown in RCT studies [75,76], suggesting that using BTV alone may have the same effect. Bifidobacteria-fermented milk (BFM) containing live bifidobacteria and Yakult strains of *Bifidobacterium breve*, *Bifidobacterium bifidum*, and *Lactobacillus acidophilus* were used in a randomized controlled trial [77], where the probiotic group had a lower clinical activity index (CAI) than the placebo group, as well as increased concentrations of SCFAs, especially fecal butyrate and propionate. Matsuoka et al. [78] did not confirm these results. The trial was discontinued due to lack of efficacy, with no differences in relapse prevention between the treated and untreated groups. Significant effect when using a probiotic strain *Lactobacillus casei* strain ACTT PTA 3945 was not proven and clinical remission was 82% in the probiotic group and 76% in the placebo group [79]. Application of *Bifidobacterium longum* 536 led to a reduction in the UC disease activity index (UCDAI) scores, the endoscopic index, the Mayo score, as well as clinical remission in 63% in the probiotic group and 52% in the placebo group [80]. The beneficial effects of the probiotic mixture Acronelle^®^, Bromatech SRL, Italy, (*Lactobacillus salivarius*, *Lactobacillus acidophilus*, and *Bifidobacterium bifidus* strain BGN4) with mesalazine in clinical and endoscopic activities in patients with UC were demonstrated by Palumbo et al. [81].

Using Bio-Three, multi-strain probiotics containing *Streptococcus faecalis*, *Clostridium butyricum*, and *Bacillus mesentericum* acchieved a remission rate of 69.5% in the probiotic group and 56.6% in the placebo group [82]. *Bifidobacterium*-3 strains improve clinical and therapeutic effect. The ulcerative colitis disease activity index was reduced in the probiotic group from 5.35 to 1.85 and in the mesalazine group from 5.41 to 3.60 [83]. A reduction in the Mayo score (clinical and endoscopic features) and histological score was found in the probiotic and in the placebo group by Oliva et al. [84]. No clinical benefit of Probio-Tec AB-25 (*L. acidophilus* La-5 and *Bifidobacterium animalis* subsp. *lactis* BB-12) compared to the placebo group was demonstrated in maintaining remission of ulcerative colitis [85]. Rectal administration of *L. casei* DG altered colonic microbiota, increased the number of *Lactobacillus* spp., decreased *Enterobacteriaceae*, TLR-4 and IL-1β, and increased mucosa IL-10. Oral administration has no effect on intestinal flora and TLR expression [86].

The most common probiotic cocktail used in the treatment of ulcerative colitis is VSL#3. This mixture contains a combination of bacteria *Lactobacillus paracasei*, *Lactobacillus plantarum*, *Lactobacillus acidophilus*, *Lactobacillus delbrueckii* subspecies *bulgaricus*, *Bifidobacterium longum*, *Bifidobacterium breve*, *Bifidobacterium infantis*, and *Streptococcus thermophilus*. Tursi et al. [87] compared VSL#3 combined with balsalazide, balsalazide alone, or mesalazine and found that the combination helped achieve remission in 85.71%, in balsalazide alone in 80.77%, and in mesalazine 72.73%. Treatment with VSL#3 in combination with anti-inflammatory drugs appears to be more effective than treatment with anti-inflammatory drugs alone. Clinical improvement and achieved remission after application of VSL#3 were also documented in another study [88]. Six other RCTs have presented beneficial results with the use of VSL#3 in the treatment of ulcerative colitis [89,90,91,92,93,94]. Zocco et al. [95] found that *Lactobacillus* GG had the same effect as mesalazine in terms of relapse rate but was more effective than mesalazine in prolonging relapse-free duration.

**Table 1:** UC—Randomized controlled trials (adults, children).

Disease Characteristics	Probiotics	Compared Groups	Results	Ref.
Mild to moderate (UCDAI) active UC	*E. coli* Nissle 1917	Probiotics vs. placebo (50% in both groups received 5-ASA)	Effective in preventing the exacerbation of IBDQ scores, clinical remission (Mayo score ≤ 2) and endoscopic remission (Mayo score = 0) in patients with mild to moderate UC	[62]
Active UC	*E. coli* Nissle 1917	Probiotics vs. placebo	No benefit in the of *E. coli* Nissle 1917 as an add-on treatment to conventional therapies for active UC. Remission reached in group *E coli* Nissle 1917 was 54% and in the group receiving placebo was 89%.	[63]
Moderate active UC	*E. coli* Nissle 1917 enema	Probiotics vs. placebo	A dose-dependent effect of rectal *E coli* Nissle 1917 compared to placebo was observed in PP (per protocol population) patients with active, mild or moderate UC, but not in the ITT (intent to treat) population. Enema with 40 mL of *E. coli* appears to be the most promising. Clinical remission (UCDAI ≤ 2) and endoscopic healing (UCDAI = 0).	[64]
Active UC	*E. coli* Nissle 1917	Probiotics vs. mesalazine (+gentamicin in both groups)	In the mesalazine group, remission was in 75% of patients compared to 68% of patients with *E. coli* Nissle 1917. Relapse was in 73% of patients in the mesalazine group and in 67% of patients inthe *E. coli* group. Treatment with *E. coli* Nissle 1917 had a similar effect to mesalazine in maintaining remission of UC.	[65]
UC in remission	*E. coli* Nissle 1917	Probiotics vs. mesalazine	Clinical active index (CAI) was not different between groups. Relapse was in 11.3% of patients in the mesalazine group and in 16.0% of patients in the *E coli*. Nissle 1917 group.	[66]
UC in remission	*E. coli* Nissle 1917	Probiotics vs. mesalazine	The per protocol (PP) analysis revealed relapse in 36.4% of patients in the *E. coli* group and in 33.9% in the mesalazine group. Adverse events were reported in 42% patients treated with *E. coli* and in 35.2% treated with mesalazine.	[67]
UC in remission—pediatric study	*E. coli* Nissle 1917	Probiotics vs. 5-ASA	Relapse rate was 25% in the *E. coli* Nissle 1917 group and 30% in the 5-ASA group. No serious adverse events were recorded. *E. coli* Nissle 1917 is effective for maintenance therapy of UC in young patients.	[68]
Mild to moderate active UC	Probiotic product (Jamieson Lab., Canada, N8W5B5) *Lactobacillus paracasei* (A234), *L. gasseri* (A237), *L. rhamnosus* (A119), *L. rhamnosus* (A139), *L. acidophillus* (A118), *L. plantarum* (A138), *L. casei* (A179), *L. reuteri* (A113), *Lactococcus lactis* (A328), *Bifidobacterium animalis* subsp. *lactis* (A026), *B. breve* (A055), *B. longum* susp. *Longum* (A027), *B.bifidus* (A 058), *B. longum* subsp. *infantis* (041)	Probiotics vs. placebo	Probiotic supplementation induced remission, lower stool frequency, and reduced the total partial Mayo score (PMS) from 3.42 to 1.33 (*p* < 0.001). Compared to the control group, the probiotic group showed a decrease in the level of C-reactive protein, IgA, an increase in the levels of hematological parameters (hematocrit, hemoglobin, RCB), and an increase in IL-10.	[30]
Mild to moderate active UC	Probiotic product (Jamieson Lab., Canada, N8W5B5) *Lactobacillus*-9 strains, *Bifidobacterium species*-5	Probiotics vs. placebo	Probiotic group had asignificantly higher score in the systemic, social, bowel, emotional, and total SIBSQ in terms of pre-treatment to post-treatment periods (*p* < 0.001).	[69]
Mild to moderate (SCCAI) active UC	Lactocare^®^ synbiotic (2 strains of *Bifidobacterium*, 4 strians of *Lactobacillus*, *Streptococcus thermophilus)* + fructo-oligosaccharides	Probiotics vs. placebo	Significant decrease was observed in the SCCAI index in the treatment group from 6.54 to 4.65 (*p* < 0.017), and in the placebo group from 5.7 to 5.21 (*p* < 0.17). Lactocare treatment lasting 5 years or more resulted in a 90.9% response rate, while treatment for less than 5 years resulted in a 47.1% response rate.	[70]
Mild to moderate active IBD	Bifico—probiotic cocktail (*Enterococcus faecalis*, *Bifidobacterium longum*, *Lactobacillus acidophilus)*	Probiotics + mesalazine vs. mesalazine	Probiotics + mesalazine improved microflora composition in IBD patients, increased count of *Bifidobacterium* and *Lactobacillus*, and reduced levels of IL-6, C-reactive protein, lactoferrin, α-1-antitrypsin, and β-2 microglobulin.	[71]
Mild to moderate active UC	*Lactobacillus acidophilus*, *L. plantarum*, *L. rhamnosus*, *L. bifidus*, *L. casei*, *Bifidobacterium infantis*	Probiotics+ 5-ASA vs. 5-ASA	Clinical remission in treated patients showed improvement in disease activity (54.9% vs. 23.5%) and histological index (82.3% vs. 41.4%).	[72]
UC and CD in remission	Synprove multi-strain (*L. rhamnosus*, *L. plantarum*, *L. acidophilus*, *Enterococcus faecium*)	Probiotics vs. placebo	Significantly reduced fecal calprotein levels in treated UC patients. No significant changes were demonstrated in CD.	[73]
Mild and moderate to severe active UC	Bifid Triple Viable (BTV, *Enterococcus faecalis*, *Bifidobacterium longum*, *L. acidophilus*)	Probiotics+ 5-ASA vs. 5-ASA	Clinical symptoms score (Mayo score) after treatment was 2.46 in the probiotic group, and 3.96 in the control group. Colon mucosa inflammation score after treatment was 0.54 in the probiotic group and 0.71, inthe control group.Colon mucosa had a decreased expression of IL-1β and an increased expression of IL-10 and IgA.	[74]
UC in remission	Bifid Triple Viable (BTV, *Enterococcus faecalis, Bifidobacterium longum*, *L. acidophilus*)	Probiotics vs. placebo	In the probiotic group, the concentrations of fecal lactobacilli and bifidobacteria were significantly increased, expression of TNF-α and IL-1β were decreased, and there was an elevated expression of IL-10. In the probiotic group, 20% of patients had a relapse during the 2 mo follow-up period, and 93% in the placebo group.	[75]
Mild to moderate (UCDAI) active UC	Bifid Triple Viabel (BTV, *Enterococcus faecalis*, *Bifidobacterium longum*, *L. acidophilus*)	Probiotics+ 5-ASA vs. 5-ASA	Clinical symptoms and UCDAI were decreased in both groups, levels of TNF-α and IL-8 were reduced and IL-10 was increased	[76]
Mild to moderate active UC	BFM (*B. breve* strain Yakult, *B. bifidum* strain Yakult, *L. acidophilus*)	Probiotics +5-ASA vs. placebo +5-ASA	CAI (clinical activity index) scoreshoweda decrease of at least three points. Clinical remission of 40% was achieved in the BFM group and 33% in the placebo group. Endoscopic activity index and histological score were reduced in the BFM group after 12 weeks of treatment. SCFA levels were increased.I in the BFM group, especially butyrate and propionate.	[77]
UC in remission	BFM bifid fermented milk (*Bifidobacterium breve* strain Yakult, *L. acidophilus*)	Probiotics vs. placebo	The study was discontinued due to lack of efficacy. Relapse-free survival did not differ between BFM and placebo groups. *Bifidobacterium* spp. were decreased before relapse.	[78]
Mild to moderate (UCDAI, TW) active UC	*L. casei* strain ACTT PTA 3945	Probiotics vs. placebo	No significant effect was observed when using the probiotics strain. Clinical remission in the probiotics group was 82% and 76% in the placebogroup. Relapse rates did not differ between the groups (14.3% vs. 26..7%).	[79]
Mild to moderate (UCDAI) active UC	*Bifidobacterium longum* 536	Probiotics vs. placebo	Clinical remission (UCDAI ≤ 2) was 63% in probiotic groups and 52% in placebo groups. Endoscopic index, Mayo score, and UCDAI score showed a significant decrease in the probiotic group but not in the placebo group.	[80]
Moderate to severe active UC (UCDAI)	*Lactobacillus salivarius, L. acidophilus, Bifidobacterium bifidus* strain BGN4	Probiotics+ 5-ASA vs. 5-ASA	There were beneficial effects of probiotics in clinical and endoscopic activities.	[81]
Inactive UC	Bio-Three tablets (*Streptococcus faecalis* T-110, *Clostridium butyricum* TO-A, *Bacillus mesentericus* TO-A)	Probiotics vs. placebo	The remission rate was 69.5% in the probiotic group and 56.6% in the placebo group at 12 mo. Cluster analysis of fecal flora are as follows—7 patients to cluster I, 32 to cluster II, and 7 to cluster III.	[82]
Active UC	*Bifidobacterium*—3 strains	Probiotics + mesalazine vs. mesalazine	Serum levels of TNF-α and IL-8 were decreased in both groups. IL-10 was increased in the group with probiotics and mesalazine. Probiotics improve clinical and therapeutic effects. UCDAI decreased in the probiotic group (from 5.36 to 1.85) and in the mesalazine group (from 5.41 to 3.60).	[83]
Mild to moderate active UC—pediatric study	*Lactobacillus reuteri* ATCC 55730	Probiotics vs. placebo	The Mayo (clinical and endoscopic features) significantly decreased in the probiotic and placebo groups. Histological score decreased only in the probiotic group. In the probiotic group, cytokine mucosal expression of IL-10 was increased, and IL-1β, TNF-α and IL-8 were decreased.	[84]
UC in remission	Probio-Tec AB-25 (*L. acidophilus* La-5 and *Bifidobacterium animalis* subsp. *lactis* BB-12)	Probiotics vs. placebo	No clinical benefit of Probio-Tec AB-25 compared to placebo was demonstrated in the maintenance of UC in remission. 25% of patients in probiotic groups maintained remission after 1 year of treatment and T 8% in placebo group.	[85]
Mild active UC	*Lactobacillus casei* DG enema and oral	Probiotics (enema, oral) + 5-ASA vs. 5-ASA	Rectal administration of *L. casei* DG altered colonic microbiota, increased the number of *Lactobacillus* spp., decreased *Enterobacteriaceae*, TLR-4 and IL-1β, and increased mucosa IL-10. Oral administration has no effect on intestinal flora and TLR expression.	[86]
Mild to moderate active UC	VSL#3 (*L. paracasei*, *L. plantarum*, *L. acidophilus*, *L. delbrueckii* subsp. *bulgaricus*, *B. longum*, *B. breve*, *B. infantis*, *Streptococcus thermophilus*)	Probiotics + balsalazide vs.balsalazide alone vs. mesalazine	The combination of VSL#3 + balsalazide is effective in the treatment of mild-to-moderate active UC an in achieving remission (85.71%), less in balsalazide alone (80.77%), and even lesss in mesalazine (72.73%).	[87]
Mild to moderate active UC	VSL#3 (*L. paracasei*, *L. plantarum*, *L. acidophilus*, *L. delbrueckii* subsp. *bulgaricus*, *B. longum*, *B. breve*, *B. infantis*, *Streptococcus thermophilus*)	Probiotics + 5-ASA vs. 5-ASA	Clinical remission in VSL#3 group was47.7% and in placebo group, 32.5%. Clinical improvement was demonstrated (reduction in UCDAI, VSL#3 vs. placebo 63.1% vs. 40.8%). Stool frequency, endoscopic scores and physician’s rate of disease activity were without differences. Mild side effects were experienced by 11.2% of patients in the VSl#3 group and 12.3% of patients in the placebo group.	[88]
Remission of chronic pouchitis in patients with ileal pouch-anal anastomosis	VSL#3 (*L. paracasei*, *L. plantarum*, *L. acidophilus*, *L. delbrueckii* subsp. *bulgaricus*, *B. longum*, *B. breve*, *B. infantis*, *Streptococcus thermophilus*)	Probiotics vs. placebo	Relapse with a 9 mo follow-up period occurred in 15% patients in the VSL#3 group, and 100% in the placebo group. The counts of fecal lactobacilli, bifidobacteria, and *S. thermophilus* significantly increased (*p* < 0.01) in the VSL#3 group.	[89]
Prevention of acute pouchitis in patients with ileal pouch-anal anastomosis	VSL#3 (*L. paracasei*, *L. plantarum*, *L. acidophilus*, *L. delbrueckii* subsp. *bulgaricus*, *B. longum*, *B. breve*, *B. infantis*, *Streptococcus thermophilus*)	Probiotics vs. placebo	Active pouchitis is defined by PDAI ≤ 7. 10% of patients treated with VSL#3 had an episode of acute pouchitis, and 40% of patients in placebo group.	[90]
UC in remission, with ATB-responsible pouchitis	VSL#3 (*L. paracasei*, *L. plantarum*, *L. acidophilus*, *L. delbrueckii* subsp. *bulgaricus*, *B. longum*, *B. breve*, *B. infantis*, *Streptococcus thermophilus*)	Probiotics vs. placebo	Remission was achieved in 85% of patients with VSL#3 and in 6% of patients with placebo. Remission (PDAI, pouchitis disease activity index) ≤ 2 + endoscopic PDAI ≤ 1.	[91]
Active UC—pediatric study	VSL#3 (*L. paracasei*, *L. plantarum*, *L. acidophilus*, *L. delbrueckii* subsp. *bulgaricus*, *B. longum*, *B. breve*, *B. infantis*, *Streptococcus thermophilus*)	Probiotics + 5-ASA vs.placebo + 5-ASA, in both groups + steroid induction	Clinical remission was achieved in 92.8% in the VSL#3 group and 36.4% in the placebo group. Relapse within 6 months of diagnosis occurred in 3 patients in the VSL#3 group and in 6 patients in the placebo group. Endoscopic and histological scores decreased more in the VSL#3 group than in the placebo group.	[92]
Mild to moderate active UC	VSL#3 (*L. paracasei*, *L. plantarum*, *L. acidophilus*, *L. delbrueckii* subsp. *bulgaricus*, *B. longum*, *B. breve*, *B. infantis*, *Streptococcus thermophilus*)	Probiotics vs. placebo	Improvement in UCDAI scores and individual symptomsoccuredat weeks 6 and 12 in the VSL#3 group compared to the placebo group. The number of patients who achieved remission in the VSL#3 group was 42.9% and in the placebo group was 18.6%.	[93]
Mild to moderate active UC	VSL#3 (*L. paracasei*, *L. plantarum*, *L. acidophilus*, *L. delbruecki* subsp. *bulgaricus*, *B. longum*, *B. breve*, *B. infantis*, *Streptococcus thermophilus*)	Probiotics vs. placebo	In the VSL#3 group, dendritic cell TLR-2 expression was reduced, the IL-10 level was increased, and IL-12p40 was decreased. Treatment of UC patients with VSL#3 favorably modulated dendritic cells, increased regulatory cytokines, and decreased pro-inflammatory cytokines and TLR expression.	[94]
UC in remission	*Lactobacillus* GG	Probiotics vs. mesalazine vs. probiotics + mesalazine	Clinical remission after 6 months of treatment was 91% in the LGG group, 87% in the mesalazine group, and 94% in the combination group. Clinical relapse rate was not significant among the three groups. Endoscopic relapse rates were not statistically significant in the three groups but increased slightly after 6 and 12 months of treatment.	[95]

Abbreviations: 5-ASA, Aminosalicylic acid; CAI, clinical activity index; SIBDQ, short inflammatory bowel disease questionnaire; PMS, partial Mayo score; PDAI, clinical disease activity index; UCDAI, ulcerative colitis disease activity index; IBDQ, inflammatory bowel disease questionnaire; SCCAI, simple clinical colitis activity index, TLR, toll-like receptor, TNF, tumor necrosis factor; IL, interleukin; IgA, immunoglobulin A; *L. Lactobacillus*; *B. Bifidobacterium*; *E. Escherichia.*

## 6. Randomized Clinical Trials on the Use of Probiotics in Crohn’s Disease

Lactobacillus rhamnosuss GG, Lactobacillus johnsoni LA1, Saccharomyces boulardii, VSL #3, Bifid Triple Viable (BTV, Enterococcus faecalis, Bifidobacterium longum, Lactobacillus acidophilus), and E. coli Nissle 1917 were the most commonly used probiotics in randomized clinical trials in patients with Crohn’s disease. Administration of Saccharomyces boulardii has been shown in clinical trials characterized as patients with CD remission [96,97,98]. Guslandi et al. [96] reported that clinical relapses occurred in 37% of patients in the mesalazine group and in 6.25 % of patients treated with the combination of mesalazine and S. boulardii. Garcia Vilela et al. [97] discovered that in patients with Saccharomyces boulardii, intestinal permeability improved according to the reduction a lactulose/mannitol ratio and altered intestinal mucosal barrier integrity. In the placebo group, the lactulose/mannitol ratio was increased. Bourreille et al. [98] reported opposite results, finding no significant differences in mean CDAI scores between groups and time to relapse between patients receiving Saccharomyces boulardii or placebo. Only one study used the probiotic Bifid Triple Viable [99]. They found that levels of inflammatory markers (C-reactive protein, TNF-α, IL-10) were lower in the treatment group than in the control group (*p* < 0.001). In both groups, levels of Lactobacilli were increased, and Enterocci with Peptococcus decreased. Probiotics with glucocorticoids reduced the incidence of infection, abdominal distension, diarrhea, and other side effects. CD patients after ileo-caecal resection after VSL#3 administration had reduced inflammatory cytokine levels compared to placebo group [100]. A pilot study was conducted in which a non-pathogenic *E. coli* Nissle 1917 strain was tested for efficacy and tolerability in maintaining remission in patients with Crohn’s disease. Reducing the risk of relapse and minimizing the need of glucocorticoids appear to be a promising approach. More placebo-controlled studies are needed to provide conclusive evidence [101].

*Lactobacillus johnsonii* LA1 was used in the two clinical studies in patients with CD [102,103]. *Lactobacillus johnsonii* was ineffective in preventing endoscopically controlled CD recurrence after bowel resection [102]. A study by Van Gosum et al. [103] demonstrated that oral intervention with *Lactobacillus johnsonii* in patients with Crohn’s disease after ileocecal resection did not prevent early recurrence.

Three studies investigated the effect of *Lactobacillus rhamosus* GG in patients with CD. Bousvaros et al. [104] followed 75 adolescent CD patients for 2 years and found that relapse was 31% in the *Lactobacillus rhamnosus* GG group and 17% in the placebo group. *Lactobacillus rhamnosus* GG as a supplement does not prolong the time to relapse in children with CD. Schultz et al. [105] investigated whether oral *Lactobacillus rhamnosus* GG could induce or maintain remission in CD. Only five patients completed the study, and the time to relapse was 16 weeks in *Lactobacillus rhamnosus* GG group and 12 weeks in the placebo group. *Lactobacillus rhamnosus* GG has shown no benefit in inducing or maintaining remission in Crohn’s disease. Even though the third study did not show a benefit of *Lactobacillus rhamnosus* GG, it did not prevent endoscopic recurrence [106].

The research highlights the importance of finding microbial biomarkers, which might serve as predictors, permitting the stratification of ulcerative colitis (UC) patients into distinct clinical entities of the UC spectrum.

Recent evidence demonstrates potential links between mitochondrial dysfunction and inflammatory bowel diseases (IBDs). In addition, bidirectional interactions between the gut microbiota and host mitochondria may modulate intestinal inflammation.

It is important to highlight that in addition to probiotic, prebiotics, synbiotics, and dietary supplements (e.g., omega-3 fatty acids) improve the integrity of tight junction, reduce systemic inflammation, and thus provide protection against IBD progression. They could open up new avenues for therapeutic interventions aimed at mitigating IBD severity in people with IBD [107,108,109].

Table 1 and Table 2 show the most important results from the studies conducted regarding the type of probiotics used and the clinical outcomes. These clinical outcomes present a challenge and an opportunity to include other probiotics, such as next-generation probiotics (NGPs), in further clinical trials. There is growing interest in NGPs, specifically *Eubacterium hallii*, *Faecalibacterium prausnitzii*, *Roseburia* spp., *Akkermansia muciniphila*, and *Bacteroides fragilis* as biotherapeutics that alter the gut microbiome and influence the development of various diseases including IBD.

**Table 2:** CD—Randomized controlled trials (adults, children).

Disease Characteristics	Probiotics	Compared Groups	Results	Ref.
CD in remission	*Saccharomyces boulardii*	Probiotics + mesalazine vs. mesalazine	Clinical relapses as assessed by CDAI > 150 points occurred in 37% of patients in the mesalazine group and in 6.25% of patients treated with the combination of mesalazine and probiotics.	[96]
CD in remission	*Saccharomyces boulardii*	Probiotics vs. placebo	In patients with *S. boulardii*, intestinal permeability improved according to a reduction of the lactulose/mannitol ratio. In the placebo group, the lactulose/mannitol ratio increased.	[97]
CD in remission	*Saccharomyces boulardii*	Probiotics vs. placebo	CD relapse was 47.5% in the *S. boulardii* group and 53.2% in the placebo group. Crohn’s disease activity index (CDAI) was not different between the groups. *S. boulardii* is safe but has no beneficial effects on CD patients in remission after steroid or salicylate therapies.	[98]
Active CD	Bifid Triple Viable (BTV, *Enterococcus faecalis*, *Bifidobacterium longum*, *L. acidophilus*)	Probiotics + glucorcorticoids + sulfasalazine vs. sulfasalazine	Levels of inflammatory markers (CRP, TNF-α, IL-10) were lower in the treatment group than in the control group (*p* < 0.01). In both groups, levels of *Lactobailli* were increased, and *Enterococci* with *Peptococcus* decreased. Probiotics with glucorticoids reduced the incidence of infection, abdominal distension, diarrhea, and other side effects.	[99]
CD after ileo-caecal resection	VSL#3 (*L. paracasei*, *L. plantarum*, *L. acidophilus*, *L. delbruecki* subsp. *bulgaricus*, *B. longum*, *B. breve*, *B. infantis*, *Streptococcus thermophilus*)	Probiotics vs. placebo	Patients in the VSL#3 group had reduced levels of inflammatory cytokine compared to the placebo group (*p* < 0.05). Major recurrencea rates at day 90 was 20.5% and 42.1% in the VSL#3 group at 365 days.	[100]
Remission of colonic CD	*E. coli* Nissle 1917	Probiotics vs. placebo	Clinical relapses were assessed by CDAI > 150 points. The application of *E. coli* Nissle 1917 reduced the risk of relapse and minimized the need for glucocorticoids.	[101]
CD after ileo-caecal resection	*Lactobacillus johnsonii* LA1	Probiotics vs. placebo	Endoscopic recurrence was 64% in the placebo group and 49% in the LA1 group at 6 mo. The distribution of the endoscopic score did not differ between groups, and LA1 did not have a sufficient effect.	[102]
CD after ileo-caecal resection	*Lactobacillus johnsonii* LA1	Probiotics vs. placebo	Severe recurrence (i3 + i4) was 21% in the LA1 group and 15% in the placebo group using ITT analysis, and according to PP analysis, it was 19% in the LA1 group and 9% in the placebo group. Clinical relapse rate (CDAI) > 70 points was 15% in the LA1 group and 13.5% in placebo group.	[103]
CD in remission-pediatric study	*Lactobacillus rhamnosus* GG	Probiotics vs. placebo	Relapse (PCDAI > 30 points) was 31% in the LGG group and 17% in the placebo group. LGG as a supplement did not prolong the time to relapse in children with CD.	[104]
CD in remission	*Lactobacillus rhamnosus* GG	Probiotics vs. placebo	Relapse was defined as an increase in CDAI > 100 points. Time to relapse was 16 weeks in LGG the group and 12 weeks in the placebo group. LGG had shown no benefit in inducing or maintaining remission in CD.	[105]
CD after ileo-caecal resection	*Lactobacillus rhamnosus* GG	Probiotics vs. placebo	Clinical recurrence (CDAI >150 points) was 16.6% in the LGG group and 10.5% in the placebo group. Endoscopic recurrence occurred in 60% in the LGG group and in the 35.3% in the placebo group. LGG dis not prevent endoscopic recurrence.	[106]

Abbreviations: CDAI, clinical disease activity index; PCDAI, pediatric Crohn’s disease activity index; *S. Saccharomyces*; CRP, C-reactive protein, TNF, tumor necrosis factor; IL, interleukin; *L. Lactobacillus*; *B. Bifidobacterium*; *E. Escherichia*.

## 7. Conclusions

In summary, IBD is a very complex disease and the direct causes and pathomechanisms are not fully understood. IBD-related conditions appears to dependd on many factors, including genetic background, environmental, immunological, and microbial factors. Despite the availability of a wide range of biological and molecular targeted therapies, the failure rate of primary and subsequent treatments of IBD remains high. The development of new therapeutic targets and calibration of current therapies is crucial to improve efficacy, safety, and tolerability. The mechanism of the therapeutic effect of probiotics in IBD is based on repair of the intestinal barrier, regulation of the balance of the intestine, and modulating the intestinal immune response. Differences in probiotic benefits between CD and UC may be due to the different extent of lesion and immune-mediated pathophysiology in the two conditions. More RCTs are needed in the future to investigate and validate the efficacy of individual probiotic strains and combined probiotic applications in IBD. The preventive use of probiotics in individuals who are susceptible to IBD should be considered and the mechanism and course of action of probiotics should also be better understood.

## Data Availability

Data will be made available upon request.
